# Supplementary material for: The Role of m6A Modification and m6A Regulators in Esophageal Cancer
Source: Cancers (Basel). 2022 Oct 20;14(20):5139. doi: 10.3390/cancers14205139 (PMC9600289; doi:10.3390/cancers14205139)
Supplement: Supplementary file 1 [file cancers-14-05139-s001.zip › cancers-1955556-supplementary.pdf]

Supplementary Table S1. Expression, clinical significance and biological function of m6A regulators in ESCA.

m6A regulator	Sample type	Methodolo gy	Expression	Role	Biological function	Referen ce
METTL3	ESCC tissue (53 pairs)	IHC	Upregulated	Oncogen e	Promote migration and invasion	[59]
	Cell (TE1, TE13, Eca109, EC1, and HEEC)	qRT-PCR and Western blot	Upregulated	Oncogen e		
METTL3	ESCA tissue (348 pairs)	ELISA	Upregulated	Oncogen e	Platinum resistance	[122]
METTL3	ESCC tissue (207 pairs)	IHC	Upregulated	Oncogen e		[123]
METTL3	Cell (Eca109, KYSE150, and HEEC)	qRT-PCR	Upregulated	Oncogen e	Promote proliferation, migration, and invasion and inhibit apoptosis	[124]
METTL3	ESCA tissue (57 pairs)	IHC	Upregulated	Oncogen e	High SUVmax	[125]
METTL3	ESCC tissue (81 pairs)	IHC	Upregulated	Oncogen e	Promote proliferation	[69]
	Cell (TE1, TE10, KYSE30, KYSE70, KYSE140, KYSE150, KYSE180, KYSE410, KYSE450, and HET1A)	qRT-PCR and Western blot	Upregulated	Oncogen e		
METTL3	ESCC tissue (53 pairs)	qRT-PCR and Western blot	Upregulated	Oncogen e	Promote proliferation, migration, and invasion and inhibit apoptosis	[126]
	Cell (TE9, Eca109, KYSE150, EC9706, and HET1A)	qRT-PCR and Western blot	Upregulated	Oncogen e		
METTL3	590 ESCC tissues and 108 adjacent normal tissues	IHC	Upregulated	Oncogen e	Promote proliferation, migration, and invasion	[60]
METTL3	ESCC tissue (66 pairs)	qRT-PCR and	Upregulated	Oncogen e	Promote proliferation	[85]

		Western blot				
METTL3	ESCA tissue (60 pairs)	qRT-PCR	Upregulated	Oncogene	Promote proliferation, EMT, migration, invasion, and metastasis	[75]
	Cell (Eca109, EC9706, KYSE150, TE1, TE10, and HEEC)	qRT-PCR	Upregulated	Oncogene		
METTL3	Cell (KYSE30,KYSE 150, TE1, Eca109, EC9706, and HET1A)	Western blot	Upregulated	Oncogene	Promote lymphangiogenesis and lymphatic metastasis	[76]
METTL3	ESCC tissue (215 pairs) ESCC tissue (58 pairs)	qRT-PCR IHC	Upregulated	Oncogene	Promote proliferation, migration, and invasion	[61]
METTL3	ESCC tissue (60 pairs)	qRT-PCR IHC Western blot	Upregulated	Oncogene	Promote proliferation, migration, and invasion	[127]
METTL3	ESCC tissue 114 Normal tissue 66	IHC	Upregulated	Oncogene	Promote invasion and metastasis	[72]
METTL3	ESCC tissue (4 pairs)	IHC Western blot	Upregulated	Oncogene	Promote migration,	128
METTL14	ESCA tissue (348 pairs)	ELISA	Upregulated	Oncogene		[122]
WTAP	ESCA tissue (40 pairs)	qRT-PCR	Upregulated	Oncogene	Promote proliferation and inhibit apoptosis	[129]
	Cell (EC9706, Eca109, KYSE150, KYSE450, TE1, TE10, and HET1A)	qRT-PCR	Upregulated	Oncogene		
WTAP	ESCC tissue (14 pairs)	qRT-PCR	Upregulated	Oncogene		[85]
WTAP	ESCC tissue (60 pairs)	qRT-PCR	Upregulated	Oncogene		[127]
RBM15	ESCC tissue (14 pairs)	qRT-PCR	Upregulated	Oncogene		[85]

KIAA1429	ESCC tissue (14 pairs)	qRT-PCR	Upregulated	Oncogene		[85]
ALKBH5	ESCA tissue (348 pairs)	ELISA	Downregulated	Tumor suppressor		[122]
ALKBH5	ESCA tissue (80 pairs)	IHC	Downregulated	Tumor suppressor		[130]
ALKBH5	ESCC tissue (60 pairs)	qRT-PCR	Downregulated	Tumor suppressor	Inhibit proliferation, migration, and invasion	[80]
ALKBH5	206 ESCC tissues and 31 adjacent normal tissues	IHC	Downregulated	Tumor suppressor	Inhibit proliferation, migration, and invasion	[131]
	ESCC tissue (20 pairs)	Western blot	Downregulated	Tumor suppressor		
ALKBH5	ESCC tissue (23 pairs)	qRT-PCR and IHC	Downregulated	Tumor suppressor	Inhibit proliferation, migration, and invasion and induce apoptosis	[86]
FTO	ESCA tissue (348 pairs)	ELISA	Downregulated	Tumor suppressor		[122]
FTO	ESCC tissue (80 pairs)	IHC	Upregulated	Oncogene	Promote proliferation and migration	[132]
FTO	ESCC tissue (44 pairs)	qRT-PCR	Upregulated	Oncogene	Promote proliferation and cell cycle progression	[83]
	Cell (KYSE150, TE1, KYSE70, KYSE450, and HET1A)	Western blot	Upregulated	Oncogene		
FTO	ESCC tissue (28 pairs)<br>ESCC tissue (2 pairs)	IHC<br>Western blot	Upregulated	Oncogene	Promote proliferation, migration, and invasion	[73]
	Cell (KYSE140, KYSE180, KYSE450 , KYSE30, KYSE150, and HEEC)	qRT-PCR<br>Western blot	Upregulated	Oncogene		
FTO	ESCA tissue (106 pairs)	IHC	Upregulated	Oncogene	Promote proliferation, migration, and stemness	[74]

YTHDF1	ESCC tissue (60 pairs)	qRT-PCR	Upregulated	Oncogene		[127]
YTHDF2	ESCC tissue (14 pairs)	Western blot	Downregulated	Tumor suppressor	Inhibit proliferation and cell cycle progression	[83]
YTHDC2	ESCC tissue (94 pairs)	RNA sequencing	Downregulated	Tumor suppressor	Inhibit proliferation	[133]
IGF2BP1	Cell (Eca109, KYSE510, KYSE150, TE1, TE10, and HET1A)	Western blot	Upregulated	Oncogene	Promote proliferation and migration and inhibit apoptosis	[134]
IGF2BP1	ESCA tissue (40 pairs)	qRT-PCR	Upregulated	Oncogene	Promote proliferation and invasion	[135]
	ESCA tissue (5 pairs)	Western blot				
IGF2BP2	ESCC tissue (93 pairs)	qRT-PCR and Western blot	Upregulated	Oncogene	Promote proliferation, migration, and invasion	[66]
HNRNPA2B1	ESCC tissue (18 pairs)	qRT-PCR	Upregulated	Oncogene	Promote proliferation, migration, and invasion	[84]
	Cell (Eca109, TE10, and HEEC)	qRT-PCR and Western blot	Upregulated	Oncogene		
HNRNPA2B1	ESCA tissue (14 pairs)	qRT-PCR	Upregulated	Oncogene	Promote proliferation	[79]
HNRNPC	ESCA tissue (80 pairs)	IHC	Upregulated	Oncogene		[130]
HNRNPC	Cell (KYSE30, TE1, and HEEC)	qRT-PCR	Upregulated	Oncogene	Promote proliferation, migration, and invasion	[136]
EIF3B	ESCC tissue (8 pairs)	Western blot	Upregulated	Oncogene	Promote cell cycle progression, proliferation, and invasion and inhibit apoptosis	[137]
	Cell (Eca109, KYSE510, and HEEC)	Western blot	Upregulated	Oncogene		
EIF3E	ESCC tissue (8 pairs)	Western blot	Upregulated	Oncogene	Promote proliferation and migration	[138]

EIF3H	ESCC tissue (79 pairs)	IHC	Upregulated	Oncogene	Promote proliferation, migration, and invasion	[139]
	Cell (KYSE30, KYSE140, KYSE150, KYSE180, KYSE410, KYSE450, KYSE510, and HET1A)	Western blot	Upregulated	Oncogene		

Supplementary Table S2. The association between expression of m6A regulators and prognosis of ESCA patients.

m6A regulator	Patients (Sample size)	Methodology	Expression	Prognosis	Reference
METTL3	ESCC (93)	IHC	High expression 42 Low expression 51	High expression; poor OS	[59]
METTL3	ESCC (207)	IHC	High expression 79 Low expression 128	High expression; poor OS and DFS	[123]
METTL3	ESCC (81)	IHC	High expression 40 Low expression 41	High expression; poor OS	[69]
METTL3	ESCC (53)	qRT-PCR and Western blot	High expression 25 Low expression 28	High expression; poor OS	[126]
METTL3	ESCC (590)	IHC		High expression; poor OS and DFS	[60]
METTL3	ESCC (215)	qRT-PCR	High expression 107 Low expression 108	High expression; poor OS	[61]
METTL3	ESCC (60)	qRT-PCR	High expression 30 Low expression 30	High expression; poor OS	[127]
METTL3	ESCC (114)	IHC	High expression 51 Low expression 63	High expression; poor OS	[72]
METTL14	ESCC (86)	IHC	High expression 30 Low expression 56	Low expression; poor OS	[77]
WTAP	ESCA (40)	qRT-PCR	High expression 20 Low expression 20	Low expression; good OS and DFS	[129]
ALKBH5	ESCA (80)	qRT-PCR	High expression 23 Low expression 57	Low expression; poor OS	[130]
ALKBH5	ESCC (177)	IHC	High expression 48 Low expression 129	High expression; poor OS	[62]
ALKBH5	ESCA (105)	IHC	Positive 61<br>Negative 44	Positive expression; good OS and DFS	[78]
FTO	ESCC (177)	IHC	High expression 89 Low expression 96	No significant difference of OS	[62]
FTO	ESCC (106)	IHC		High expression; poor OS	[74
IGF2BP3	ESCC (177)<br>(surgery alone 72; adjuvant group 105)	IHC	High expression 122 (surgery alone 47; adjuvant group 75)<br>Low expression 55 (surgery alone 25; adjuvant group 30)	Surgery alone:<br>High expression; poor OS, DSS, and DFS	[154]
IGF2BP3	ESCC (191)	IHC	Positive 113 Negative 78	Positive expression; poor OS and RFS	[140]
HNRNPA2B1	ESCC (106)	IHC	Positive 67<br>Negative 39	Positive expression; poor OS	[79]

HNRNPC	ESCA (80)	qRT-PCR	High expression 66 Low expression 14	High expression; poor OS	[130]
EIF3B	ESCC (154)	IHC	High expression 79 Low expression 75	High expression; poor OS and DFS	[137]
EIF3E	ESCC (241)	IHC	High expression 218 Low expression 23	No significant difference of OS and DFS	[138]

overall survival (OS), disease-specific survival (DSS), disease-free survival (DFS), recurrence-free survival (RFS).
